# RBP-Tar – a searchable database for experimental RBP binding sites

**DOI:** 10.12688/f1000research.131014.3

**Published:** 2024-11-25

**Authors:** Katarina Gresova, Tomas Racek, Vlastimil Martinek, David Cechak, Radka Svobodova, Panagiotis Alexiou

**Affiliations:** 1 National Centre for Biomolecular Research, Faculty of Science, Masaryk University, Brno, Czech Republic; 2 Central European Institute of Technology (CEITEC), Masaryk University, Brno, Czech Republic; 3 Centre for Molecular Medicine & Biobanking, University of Malta, Msida, Malta; 4 Department of Applied Biomedical science, University of Malta, Msida, Malta

**Keywords:** RNA Binding Proteins, CLIP, RBP, Web-server

## Abstract

**Background:**

RNA-binding proteins (RBPs) play a critical role in regulating gene expression by binding to specific sites on RNA molecules. Identifying these binding sites is crucial for understanding the many functions of RBPs in cellular function, development and disease. Current experimental methods for identifying RBP binding sites, such as ultra-violet (UV) crosslinking and immunoprecipitation (CLIP), and especially the enhanced CLIP (eCLIP) protocol, were developed to identify authentic RBP binding sites experimentally.

**Methods:**

To make this data more accessible to the scientific community, we have developed RBP-Tar ( https://ncbr.muni.cz/RBP-Tar ), a web server and database that utilises eCLIP data for 167 RBPs mapped on the human genome. The web server allows researchers to easily search and retrieve binding site information by genomic location and RBP name.

**Use case:**

Researchers can produce lists of all known RBP binding sites on a gene of interest, or produce lists of binding sites for one RBP on different genomic loci.

**Conclusions:**

Our future goal is to continue to populate the web server with additional experimental datasets from CLIP experiments as they become available and processed, making it an increasingly valuable resource for the scientific community.

## Introduction

RNA binding proteins (RBPs) are key players in a broad spectrum of RNA regulation, including all stages of the RNA lifecycle ( Gerstberger *et al*. 2014; Gebauer *et al.* 2021). Eukaryotic genomes typically encode hundreds of RBPs. For example, over 1500 human RBPs involved in the maturation, transport, stability and translation of coding and non-coding RNA were recently characterised and manually curated ( Gerstberger *et al.* 2014). Each RBP can typically target hundreds of RNAs in a complex coordinated fashion ( Hogan *et al.* 2008). The general transcriptomic locations of thousands of RNA binding sites corresponding to hundreds of RBPs have been identified using a family of experimental techniques based on RBP CrossLinking, ImmunoPrecipitation and Sequencing (CLIP-Seq) ( Chi *et al.* 2009; Licatalosi *et al.* 2008; Moore *et al.* 2014; Hafner *et al.* 2010). A thorough exploration of tens of RBPs binding characteristics in vitro has shown that RBPs can differentiate their binding sites with context preferences beyond narrowly defined binding sequence motif and secondary structure often involving complex binding configurations ( Dominguez *et al.* 2018). Among the experimental techniques available to date, the enhanced CLIP (eCLIP) protocol (Van Nostrand *et al.* 2016) is particularly important, as it significantly reduces required amplification and increases specificity in identifying authentic binding sites. This improves the efficiency and accuracy of RBP binding site identification and allows for a deeper understanding of the role of RBPs in gene regulation.

The availability of a large amount of data points produced from the same experimental technique can be very beneficial for applications such as machine learning, as it allows researchers to train and test models with more confidence. Here we present RBP-Tar ( Tomáš Raček 2023), a centralised and searchable database of experimentally identified RBP binding sites that can significantly facilitate the study of the RBP mediated gene regulation. Using RBP-Tar, researchers can quickly and cleanly retrieve RBP binding sites constrained by both genomic location and associated RBP for hundreds of RBPs.

## Methods

### Implementation

**Pipeline for reproducible data download and annotation**

We have developed a reproducible and easy-to-use pipeline for downloading and annotating RBP eCLIP data from the ENCODE ( Luo *et al*. 2020) database.

Metadata for eCLIP experiments, as well as additional files containing genomic coordinates are downloaded from the ENCODE database with the following parameters:

*“status= released&*

*internal_tags=ENCORE&*

*assay_title=eCLIP&*

*biosample_ontology.term_name=K562&*

*biosample_ontology.term_name=HepG2&*

*files.file_type=bed+narrowPeak&*

*type=Experiment*

*files.analyses.status=released”.*

Following the download, information about the chromosome, start position, end position, strand and p values of reads are extracted for each RBP binding site. Binding sites are filtered by length, excluding ones shorter than 20 and longer than 100 nucleotides. As a last step, genomic sequences of binding sites are retrieved.

The described pipeline is implemented as a set of python scripts and is freely available at GitHub.

Using this pipeline, data for 168 RBPs on two cell lines (K562, HepG2) were downloaded. In total, 42 MB of data, representing more than 400 thousand binding sites, were thus processed.

**Web server**

Here we present RBP-Tar, a web server that can access the above-curated dataset of RBP binding sites ( https://rbp-tar.biodata.ceitec.cz/) and was built with Python (RRID:SCR_008394), the web development framework Flask, and a simple SQLite database (RRID:SCR_017672). The application’s source codes can be found on the project’s GitHub page, along with the requirements and instructions for the deployment if a user wants to run the application locally.

The web user interface allows searching and filtering based on the start and end position of the binding site, strand, chromosome, p value and protein name. It offers the download of the filtered data based on the search done by the user. Due to the size of the dataset, the view is limited to 10,000 results. However, the whole dataset can be conveniently downloaded as a gzipped CSV (14 MB) ( https://rbp-tar.biodata.ceitec.cz/download_all).

### Operation

The RBP-Tar web server can take as input any of the following user-provided parameters: [Start min, Start max] denote the limits of the low genomic coordinate of the locus of interest. Similarly, [End min, End max] denote the limits of the high coordinate. [Strand] and [Chromosome] can be used to narrow down the search to only one strand and a specific chromosome and [-log10(p-value) min, -log10(p-value) max] allows for filtering based on the significance of binding sites. Using these combinations of parameters, a user can easily search for binding sites on their favourite gene, exon, or even a whole chromosome. The last parameter is [Protein name], which brings out a drop-down menu of all the RBPs in the database. If this parameter is not set, all RBPs are queried.

Results are shown as a table with the [Chromosome, Start, End, Strand] genomic location of the binding site, followed by the [-log10(p-value)] p-value of the binding site, [Protein name] of the associated RBP and the [Sequence] of the binding site contains the genomic sequence of the binding site. Results can be seen online in a table format or downloaded as a CSV file with one button click. We expect most users to download the results and use them for further downstream analyses.

## Use cases

### Use case 1: all known RBP binding sites on the gene of interest

**Figure 1.**
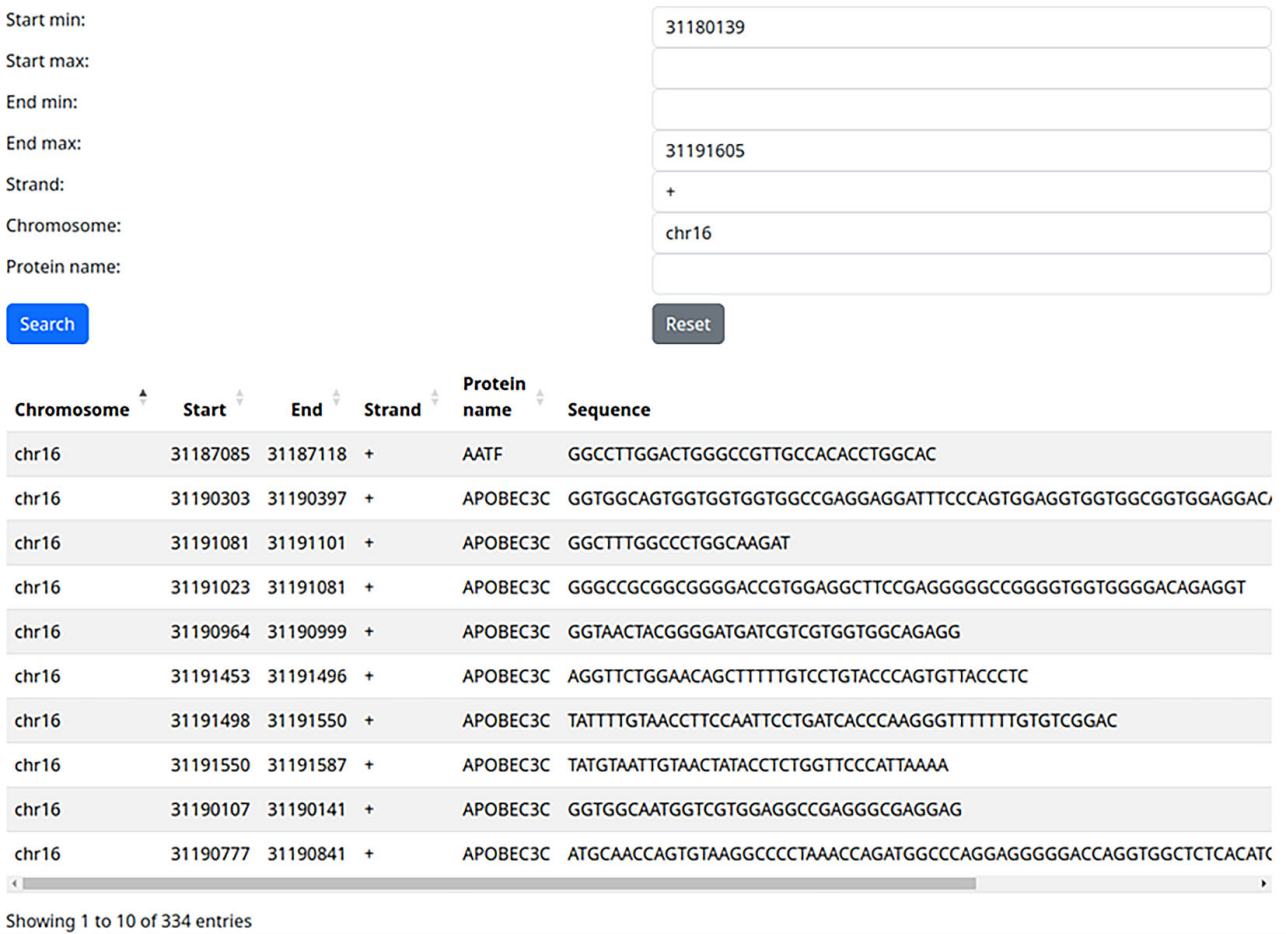
Extracting binding sites of all RNA-binding proteins on one locus (use case 1).

The first and potentially most common use case would be the query of all known RBP binding sites on a gene of interest. For example, we can query our web server with the coordinates of the Fused in Sarcoma (FUS) gene (chr16: 31180139-31191605, +) and leave the protein field empty. After this search, all 345 known RBP binding sites on this gene are returned and can be easily downloaded in a CSV file for further analysis ( Figure 1).

**Figure 2.**
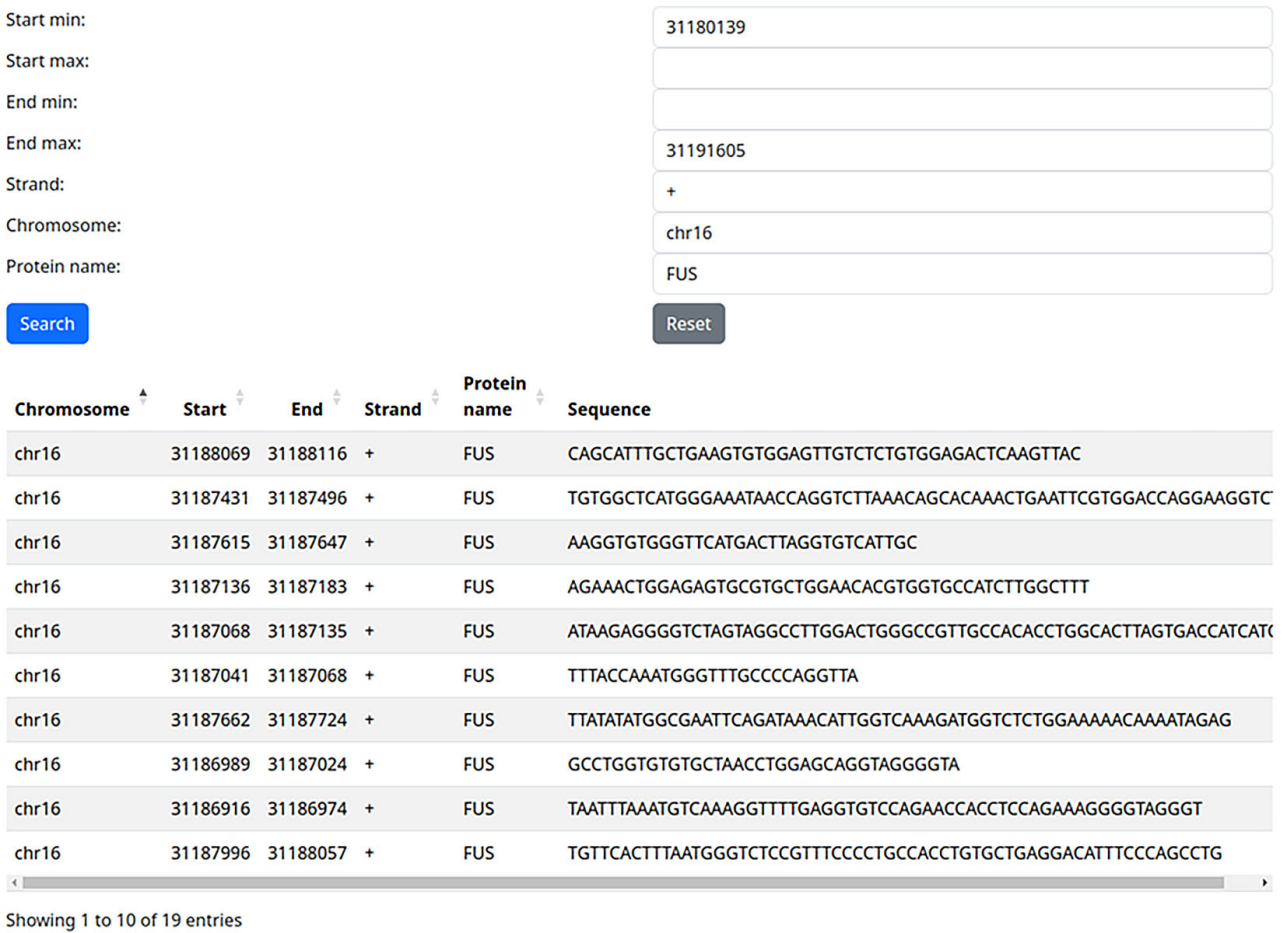
Fused in Sarcoma (FUS) self-targeting identified by filtering by gene location and protein name.

In fact, the gene we used encodes the RBP FUS, which plays important roles, among others, in neurodegeneration and cancer progression. We can use the filter [Protein Name] to identify the potential self-targeting of FUS on itself. Indeed, we can thus identify 19 potential FUS self-targeting binding sites identified *via* eCLIP ( Figure 2). Of course, the biological relevance of this type of finding is left to the users, as is the further validation of high-throughput derived RBP binding sites.

### Use case 2: training/testing dataset for one RBP

**Figure 3.**
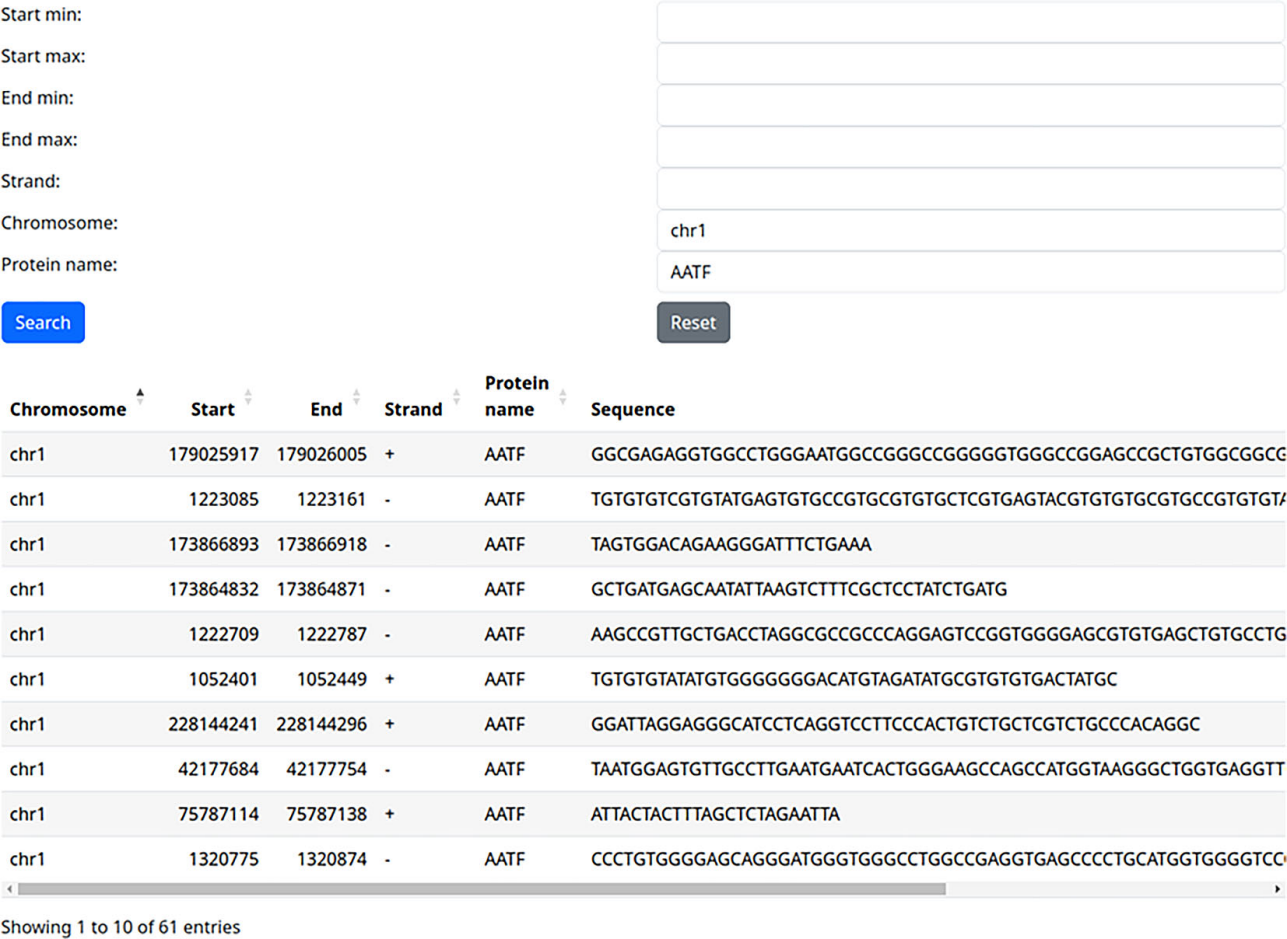
Extracting all binding sites of a single protein on a single chromosome (use case 2).

A potential user may want to develop an RBP binding site machine learning tool that would be able to predict binding sites based on a sequence. It is important to make sure that training and testing sets for their machine learning method are not overlapping. Using our web server, they can download all binding sites for a specific RBP, for example, on chromosome 1, and use them as a training set ( Figure 3). Then, do the same for chromosome 2 and use it as an independent testing set, thus ensuring that the training and testing sets do not overlap.

## Conclusion

Recent advancements in experimental techniques, such as eCLIP, have greatly expanded our understanding of RBP binding preferences and their role in gene regulation. The development of centralised and searchable databases of experimentally identified RBP binding sites allows researchers to access and analyse the binding preferences of RBPs easily. This information can be used to identify known binding sites on genes of interest and aid in training machine-learning models for RBP binding site prediction. This paper presents RBP-Tar, a centralised web server that can retrieve RBP Target sites with location and RBP constraints. RBP-Tar has been designed to be easily accessible by non-experts. It is still confined to a single source of data, which is helpful for avoiding experimental design effects, but makes its scope limited. We plan to expand the web server with other sources of data, as well as ways for the user to be able to take into account provenance and experimental variation.

## Data Availability

All data used in RBP-Tar has been downloaded from ENCODE and Ensembl projects in November 2024. RBP eCLIP metadata were downloaded from https://www.encodeproject.org/metadata/?status=released&internal_tags=ENCORE &assay_title=eCLIP&biosample_ontology.term_name=K562&biosample_ontology.term_name=HepG2&files.file_type= bed+narrowPeak&type=Experiment&files.analyses.status=released. A list of all downloaded files containing genomic coordinates can be found here: https://github.com/ML-Bioinfo-CEITEC/rbp_encode_eclip/blob/main/csv/coord_links.txt. The reference genome was downloaded from http://ftp.ensembl.org/pub/release-97/fasta/homo_sapiens/dna/Homo_sapiens.GRCh38.dna.toplevel.fa.gz Columns ‘chr’, ‘start’, ‘end’, ‘strand’ and ‘pValue’ from downloaded files containing genomic coordinates can be found here: https://github.com/ML-Bioinfo-CEITEC/rbp_encode_eclip/tree/main/csv. The whole dataset (curated and including sequence) can be downloaded from https://rbp-tar.biodata.ceitec.cz/ as a gzipped CSV (18 MB) ( https://rbp-tar.biodata.ceitec.cz//download_all).

## Software availability

### RBP-Tar

**Software:** https://rbp-tar.biodata.ceitec.cz/

**Source code:** https://github.com/sb-ncbr/RBP-Tar/releases/tag/v1.0.2

**Archived source code at the time of publication:** https://doi.org/10.5281/zenodo.14135388 ( Raček 2024)

**License:** MIT

### eCLIP RBP

**Source code:** https://github.com/ML-Bioinfo-CEITEC/rbp_encode_eclip/tree/v1.0.3

**Archived source code at the time of publication:** https://doi.org/10.5281/zenodo.14082502 ( Grešová & Raček 2024)

**License:** Apache 2.0
