# Supplementary material for: RBP-Tar – a searchable database for experimental RBP binding sites
Source: F1000Res. 2024 Nov 25;12:755. Originally published 2023 Jun 27. [Version 3] doi: 10.12688/f1000research.131014.3 (PMC11795353; doi:10.12688/f1000research.131014.3)
Supplement: Peer review history

# Reviewer report: Reviewer response for version 3

**DOI:** 10.5256/f1000research.175053.r354325

**Published:** 2025-01-16

**Authors:** Zhi-Ping Liu

**Affiliations:** 1 Shandong University, Jinan, Shandong, China

In this paper, the authors present the development of RBP-Tar, a searchable database and web server dedicated to experimental RNA-binding protein (RBP) binding sites. RBP-Tar currently focuses on eCLIP data for 167 RBPs mapped on the human genome, though its scope has the potential to be expanded to include a broader range of CLIP data to enhance its utility.

 To address the current limitation of focusing solely on eCLIP data, the authors suggest exploring the inclusion of other CLIP data types in future updates. Additionally, to clarify the purpose and content of this paper, the authors recommend refining the title and abstract to more accurately reflect the scope and contributions of their work. Furthermore, to provide context for their work, the authors need include a discussion of existing databases similar to RBP-Tar. This section should summarize the features and specificity of RBP-Tar compared to these existing resources. If possible, a comparison of the overlapping information with these databases would be beneficial to highlight the unique aspects of RBP-Tar. For the web server component of RBP-Tar, the authors need consider incorporating submission and download options for original data and query results. These features would enhance the usability and accessibility of the server, making it a more comprehensive resource for researchers studying RNA-binding proteins.

Are the conclusions about the tool and its performance adequately supported by the findings presented in the article?

Yes

Is the rationale for developing the new software tool clearly explained?

Yes

Is the description of the software tool technically sound?

Yes

Are sufficient details of the code, methods and analysis (if applicable) provided to allow replication of the software development and its use by others?

Partly

Is sufficient information provided to allow interpretation of the expected output datasets and any results generated using the tool?

Yes

Reviewer Expertise:

Bioinformatics, Systems Biology.

I confirm that I have read this submission and believe that I have an appropriate level of expertise to confirm that it is of an acceptable scientific standard.

# Reviewer report: Reviewer response for version 3

**DOI:** 10.5256/f1000research.175053.r354315

**Published:** 2025-01-16

**Authors:** M. J. Nishanth

**Affiliations:** 1 St Joseph’s University, Bengaluru, India

The authors have developed RBP-Tar, a database and tool that can be used to identify the known target motifs of RBPs on human genes or chromosomal loci. This would be a useful tool for scientific community to identify RBP target motifs. Following are the technical queries/suggestions for the authors:

 1. The authors state that the target motifs < 20 bases in length or >100 bases are excluded from the result-list. Why are the motifs < 20 bases excluded? The rationale for this parameter needs to be given.

 2. It may be beneficial to add an 'evidence' column in the result/output table. This column could have the reference of the primary study/experiment which forms the evidence for the particular target site being displayed in the result table. This would be helpful for the users if further validation of the target motifs is needed.

 3. The authors could consider adding a summary of the existing software tools in the realm of RBP target motif identification and related bioinformatic analyses. They could elaborate on the utility of RBP-Tar in the context of the existing software tools, which would give a broader perspective of the usefulness of RBP-Tar. A few of the existing relevant tools that could be discussed: RBP Map (https://rbpmap.technion.ac.il/), Transite (https://transite.mit.edu/), oRNAment (https://rnabiology.ircm.qc.ca/oRNAment/), and ATtRACT (https://attract.cnic.es/).

 4. The authors need to detail the statistical tests and analyses on which the p-value calculations are based.

Are the conclusions about the tool and its performance adequately supported by the findings presented in the article?

Yes

Is the rationale for developing the new software tool clearly explained?

Partly

Is the description of the software tool technically sound?

Yes

Are sufficient details of the code, methods and analysis (if applicable) provided to allow replication of the software development and its use by others?

Yes

Is sufficient information provided to allow interpretation of the expected output datasets and any results generated using the tool?

Yes

Reviewer Expertise:

Molecular biology, gene regulation

I confirm that I have read this submission and believe that I have an appropriate level of expertise to confirm that it is of an acceptable scientific standard, however I have significant reservations, as outlined above.

# Reviewer report: Reviewer response for version 3

**DOI:** 10.5256/f1000research.175053.r349169

**Published:** 2025-01-10

**Authors:** Eric Van Nostrand

**Affiliations:** 1 Baylor College of Medicine, Houston, TX, USA

This article by Gresova et al build a searchable web tool for the ENCODE eCLIP data that profiles RNA binding protein interactions. 

 Since this is a named review, I can specifically comment - as one other reviewer notes, some of the filtering choices here are done without really justifying why they are appropriate - i.e., why are peaks shorter than 20 or longer than 100nt excluded? That's something that should either be justified with analysis showing that it improves signal-to-noise, or not done. (In particular, the CLIP per peak caller used in ENCODE tends to err on the side of calling short peaks, so I highly suspect filtering peaks shorter than 20 is removing a significant amount of real signal)

 Similarly, we reported the fold-enrichment versus input because we found that to often be more informative than p-value (which is often more a function of transcript abundance rather than binding signal), but that is not reported here for an undescribed reason.

 Since the entire website is based off of the ENCODE eCLIP data, it seems appropriate to cite that manuscript (Van Nostrand EL, et al., 2020 - PMID 32728246 [Ref-1]) rather than the broader ENCODE overview Luo et al publication.

 If the idea is to make it extremely user-friendly, it seems like having an ability to copy-paste a UCSC browser-style region (e.g. "chr1:xxxx-yyyy") would make it simpler to use

Are the conclusions about the tool and its performance adequately supported by the findings presented in the article?

Yes

Is the rationale for developing the new software tool clearly explained?

Partly

Is the description of the software tool technically sound?

Yes

Are sufficient details of the code, methods and analysis (if applicable) provided to allow replication of the software development and its use by others?

Yes

Is sufficient information provided to allow interpretation of the expected output datasets and any results generated using the tool?

Yes

Reviewer Expertise:

eCLIP, RNA binding protein studies

I confirm that I have read this submission and believe that I have an appropriate level of expertise to confirm that it is of an acceptable scientific standard, however I have significant reservations, as outlined above.

## References

[1] : A large-scale binding and functional map of human RNA-binding proteins. *Nature* .2020;583(7818) : 10.1038/s41586-020-2077-3 711-719 10.1038/s41586-020-2077-3 32728246 PMC7410833

# Reviewer report: Reviewer response for version 3

**DOI:** 10.5256/f1000research.175053.r349176

**Published:** 2025-01-09

**Authors:** Michiel Stock

**Affiliations:** 1 Ghent University, Ghent, Belgium

This article presents RBP-tar, a small database with a web interface that enables users to access RNA-binding proteins in humans. Users can select regions on different chromosomes and view a list of all binding proteins. Additionally, there are various filter options, such as filtering by protein name for self-binding. Users can also directly download a CSV file containing the entire database, which I always appreciate. The database is designed to facilitate the development of machine learning models.

 The short article appears to be well explained. However, I find one aspect lacking: the comparison and relationship with similar databases, such as RBP2GO or EuRBPDB. This should be addressed in a designated section.

 I would also like some information on how this will be maintained. Will more RBPs be added?

 Furthermore, I would appreciate some additional clarification regarding what the p-values refer to.

 minor:  

 - "p values" => "p-values" (clarify what the p-values are for).  

 - what is exactly the " [-log10(p-value)] p-value of the binding site"?

Are the conclusions about the tool and its performance adequately supported by the findings presented in the article?

Partly

Is the rationale for developing the new software tool clearly explained?

Partly

Is the description of the software tool technically sound?

Yes

Are sufficient details of the code, methods and analysis (if applicable) provided to allow replication of the software development and its use by others?

Yes

Is sufficient information provided to allow interpretation of the expected output datasets and any results generated using the tool?

Yes

Reviewer Expertise:

Bioinformatics and machine learning.

I confirm that I have read this submission and believe that I have an appropriate level of expertise to confirm that it is of an acceptable scientific standard, however I have significant reservations, as outlined above.

# Reviewer report: Reviewer response for version 2

**DOI:** 10.5256/f1000research.169056.r313335

**Published:** 2024-09-05

**Authors:** Clifford A Meyer

**Affiliations:** 1 Harvard T.H. Chan School of Public Health, Dana-Farber Cancer Institute, Boston, USA

The revision is not satisfactory as most of the reviewers' suggestions for improving the website were not implemented. We hope that these will be implemented in future versions.

Are the conclusions about the tool and its performance adequately supported by the findings presented in the article?

Yes

Is the rationale for developing the new software tool clearly explained?

Yes

Is the description of the software tool technically sound?

Yes

Are sufficient details of the code, methods and analysis (if applicable) provided to allow replication of the software development and its use by others?

Partly

Is sufficient information provided to allow interpretation of the expected output datasets and any results generated using the tool?

No

Reviewer Expertise:

Epigenetics, Chromatin analysis, Gene regulation, Single cell

I confirm that I have read this submission and believe that I have an appropriate level of expertise to confirm that it is of an acceptable scientific standard, however I have significant reservations, as outlined above.

# Response

**Published:** 2024-11-19

**Authors:** Panagiotis Alexiou

**Affiliations:** University of Malta, Malta

Thank you for the continued feedback on our manuscript and webserver resource. We appreciate the reviewer’s commitment to ensuring that the resource becomes more robust and user-friendly. In response to the earlier suggestions, we have implemented the following improvements in our webserver, as outlined below:

1. Inclusion of p-values: We have added a p-value column to our data, providing an additional layer of statistical context to aid users in evaluating the significance of RNA binding protein binding sites.
2. Filtering and sorting functionality by p-value: To enhance usability, we have introduced the functionality to filter and sort binding site data based on p-values. This improvement allows users to more easily interpret and prioritize results according to their significance.

We recognize that many of the enhancements suggested in previous rounds, such as expanding the dataset scope and incorporating broader quality control metrics, require a significant overhaul and are part of our longer-term development roadmap.

 Our current updates reflect initial, impactful steps toward addressing reviewer concerns, and we remain committed to further expanding the features and datasets in future versions.

# Reviewer report: Reviewer response for version 2

**DOI:** 10.5256/f1000research.169056.r313334

**Published:** 2024-08-23

**Authors:** Xiaoyong Pan

**Affiliations:** 1 Shanghai Jiao Tong University, Shanghai, Shanghai, China

The authors addressed my comments.

Are the conclusions about the tool and its performance adequately supported by the findings presented in the article?

Partly

Is the rationale for developing the new software tool clearly explained?

Yes

Is the description of the software tool technically sound?

Yes

Are sufficient details of the code, methods and analysis (if applicable) provided to allow replication of the software development and its use by others?

Partly

Is sufficient information provided to allow interpretation of the expected output datasets and any results generated using the tool?

Partly

Reviewer Expertise:

NA

I confirm that I have read this submission and believe that I have an appropriate level of expertise to confirm that it is of an acceptable scientific standard.

# Reviewer report: Reviewer response for version 1

**DOI:** 10.5256/f1000research.143817.r224195

**Published:** 2023-12-18

**Authors:** Clifford A Meyer

**Affiliations:** 1 Harvard T.H. Chan School of Public Health, Dana-Farber Cancer Institute, Boston, USA

This paper describes a database of RNA binding protein binding sites based on 2 ENCODE cell lines. The website allows uses to search for data by querying a gene or genomic interval.

 The scope of the database is very limited. It is not clear why the data is restricted to eCLIP data and not CLIP data in general. Although the authors make the argument that eCLIP data is of good quality, quality will vary from sample to sample and there are likely to be high quality CLIP data sets as well as low quality eCLIP ones.

 It appears that the processed peaks are downloaded from ENCODE, but the methods used by ENCODE to identify these peaks are not described, and statistics such as p-values, false discovery rates and enrichment relative to background are not provided. Quality controls are also not provided, so it is not possible to know if the experiment worked. 

 As processed RBP data is downloaded from ENCODE  this resource does not add much to what is already available on the ENCODE website. A resource that processed the raw read data from other data resources, such as GEO, would be more valuable. There are over 2,000 eCLIP samples and over 6,000 CLIP samples in GEO.

 The search features are useful, although it is hard to interpret the results. Some ranking of the results from most significant to least is needed. It would also be helpful to enable filtering based on some binding sites statistics.

 Overall, the resource is limited in terms of the data included and the website features. It would be helpful to include quality control metrics to let users know which samples are more informative.

Are the conclusions about the tool and its performance adequately supported by the findings presented in the article?

Yes

Is the rationale for developing the new software tool clearly explained?

Yes

Is the description of the software tool technically sound?

Yes

Are sufficient details of the code, methods and analysis (if applicable) provided to allow replication of the software development and its use by others?

Partly

Is sufficient information provided to allow interpretation of the expected output datasets and any results generated using the tool?

No

Reviewer Expertise:

Epigenetics, Chromatin analysis, Gene regulation, Single cell

I confirm that I have read this submission and believe that I have an appropriate level of expertise to confirm that it is of an acceptable scientific standard, however I have significant reservations, as outlined above.

# Response

**Published:** 2024-07-11

**Authors:** Panagiotis Alexiou

**Affiliations:** University of Malta, Malta

*This paper describes a database of RNA binding protein binding sites based on 2 ENCODE cell lines. The website allows uses to search for data by querying a gene or genomic interval.*

*The scope of the database is very limited. It is not clear why the data is restricted to eCLIP data and not CLIP data in general. Although the authors make the argument that eCLIP data is of good quality, quality will vary from sample to sample and there are likely to be high quality CLIP data sets as well as low quality eCLIP ones.*

 We agree that the scope of the database is limited at this point and we aim to widen it in the future updates of the app. ENCODE/ENCORE has a comprehensive eCLIP experimental list, and only 3 iCLIP libraries ( https://www.encodeproject.org/encore-matrix/?type=Experiment&status=released&internal_tags=ENCORE ). We chose eCLIP to keep the experimental procedure more consistent in case of different methods introducing different biases. In a future version, we will explore using different datasets from multiple sources. 

*It appears that the processed peaks are downloaded from ENCODE, but the methods used by ENCODE to identify these peaks are not described, and statistics such as p-values, false discovery rates and enrichment relative to background are not provided. Quality controls are also not provided, so it is not possible to know if the experiment worked.* 

 Methods used by ENCODE to identify peaks from eCLIP data are described here: https://www.encodeproject.org/eclip/ while the detailed description of data processing pipeline can be downloaded from here: https://www.encodeproject.org/documents/739ca190-8d43-4a68-90ce-1a0ddfffc6fd/@@download/attachment/eCLIP_analysisSOP_v2.2.pdf . This pipeline ensures that only high-quality peaks are identified.

*As processed RBP data is downloaded from ENCODE  this resource does not add much to what is already available on the ENCODE website. A resource that processed the raw read data from other data resources, such as GEO, would be more valuable. There are over 2,000 eCLIP samples and over 6,000 CLIP samples in GEO.*

 We agree that a resource that processed the raw read data from other data resources, such as GEO, would be more valuable, we will consider adding other data resources in the future updates of the webserver. The addition of our app to what is already available on the ENCODE website is advanced filtering of binding sites based on the genome position.

# Reviewer report: Reviewer response for version 1

**DOI:** 10.5256/f1000research.143817.r224199

**Published:** 2023-12-18

**Authors:** Xiaoyong Pan

**Affiliations:** 1 Shanghai Jiao Tong University, Shanghai, Shanghai, China

1. ​​​​​In the paper, the author claim they download narrowPeak metadata from https://www.encodeproject.org/metadata/?status=released&internal_tags=ENCORE&assay_title=eCLIP&biosample_ontology.term_name=K562&biosample_ontology.term_name=HepG2&files.file_type=bed+narrowPeak&type=Experiment&files.analyses.status=released&files.preferred_default=true，and the link summary is provided in the file https://github.com/ML-Bioinfo-CEITEC/rbp_encode_eclip/blob/main/csv/coord_links.txt. In the ENCODE dataset, the majority of RNA-binding proteins (RBPs) have biological replicate(s) or technical replicate(s), with each replicate having an associated narrowPeak file. Additionally, there is another merged peak file containing reproducible peaks as determined by entropy-ordered peaks between two replicates. However, it is observed that only one file per RBP from K562 or HepG2, which is both the merged peak file. For eCLIP experiment on K562 against QKI, I just find ENCFF190XSX that is from replicate 1. A careful examination of the data is recommended to ensure accuracy and completeness.
2. After narrowPeak download, the author filter the peak by length, excluding ones shorter than 20 and longer than 100 nucleotides. It's important to note that must short (rarely more than 4–6-nucleotide-long)  sequence elements within RNA targets that are recognized and bound by RNA-binding proteins (Gerstberger, S et al. (2014 ^1^). In my perspective, employing a filter based on fold change and p-value, in accordance with the eCLIP-seq Processing Pipeline of ENCODE ( https://www.encodeproject.org/documents/739ca190-8d43-4a68-90ce-1a0ddfffc6fd/@@download/attachment/eCLIP_analysisSOP_v2.2.pdf) is powerful.
3. If this job is for the benefit of biologists, why not use transcripts as a reference? Because RBP binds to transcripts. And it's too simplistic for systems biologists, the author can refer to the POSTAR3 data.

Are the conclusions about the tool and its performance adequately supported by the findings presented in the article?

Partly

Is the rationale for developing the new software tool clearly explained?

Yes

Is the description of the software tool technically sound?

Yes

Are sufficient details of the code, methods and analysis (if applicable) provided to allow replication of the software development and its use by others?

Partly

Is sufficient information provided to allow interpretation of the expected output datasets and any results generated using the tool?

Partly

Reviewer Expertise:

The authors need solve some issues before published

I confirm that I have read this submission and believe that I have an appropriate level of expertise to confirm that it is of an acceptable scientific standard, however I have significant reservations, as outlined above.

## References

[1] : A census of human RNA-binding proteins. *Nat Rev Genet* .2014;15(12) : 10.1038/nrg3813 829-45 10.1038/nrg3813 25365966 PMC11148870

# Response

**Published:** 2024-07-11

**Authors:** Panagiotis Alexiou

**Affiliations:** University of Malta, Malta

**Reviewer Comment:**

*​​​​​In the paper, the author claim they download narrowPeak metadata from https://www.encodeproject.org/metadata/?status=released&internal_tags=ENCORE&assay_title=eCLIP&biosample_ontology.term_name=K562&biosample_ontology.term_name=HepG2&files.file_type=bed+narrowPeak&type=Experiment&files.analyses.status=released&files.preferred_default=true ，and the link summary is provided in the file https://github.com/ML-Bioinfo-CEITEC/rbp_encode_eclip/blob/main/csv/coord_links.txt . In the ENCODE dataset, the majority of RNA-binding proteins (RBPs) have biological replicate(s) or technical replicate(s), with each replicate having an associated narrowPeak file. Additionally, there is another merged peak file containing reproducible peaks as determined by entropy-ordered peaks between two replicates. However, it is observed that only one file per RBP from K562 or HepG2, which is both the merged peak file. For eCLIP experiment on K562 against QKI, I just find ENCFF190XSX that is from replicate 1. A careful examination of the data is recommended to ensure accuracy and completeness.*

***Author Response:*** We would like to thank the reviewer for the insightful finding. We have carefully examined the downloaded metadata and adjusted the processing pipeline to ensure the merged peak file is used for every RBP. In the methods, section “Pipeline for reproducible data download and annotation”, we have added the parameter “files.analyses.status=released” that is ensuring all available files are downloaded. Updated data have been uploaded to the RBP-Tar webserver and the manuscript has been updated with the current number of binding sites and size of files. We have provided new figures with up-to-date views of the web server.

**Reviewer Comment:**

*After narrowPeak download, the author filter the peak by length, excluding ones shorter than 20 and longer than 100 nucleotides. It's important to note that must short (rarely more than 4–6-nucleotide-long)  sequence elements within RNA targets that are recognized and bound by RNA-binding proteins (Gerstberger, S et al. (2014 1 ). In my perspective, employing a filter based on fold change and p-value, in accordance with the eCLIP-seq Processing Pipeline of ENCODE ( https://www.encodeproject.org/documents/739ca190-8d43-4a68-90ce-1a0ddfffc6fd/@@download/attachment/eCLIP_analysisSOP_v2.2.pdf) is powerful.*

***Author Response: *** We appreciate the reviewer's perspective on filtering criteria. Our decision to filter based on length aimed to exclude potential artifacts and prioritize biologically relevant binding sites. While fold change and p-value are valuable parameters, our focus was on ensuring the inclusion of robust binding sites within the specified length range. We will consider incorporating additional filtering parameters in future iterations leading to a new version of the web server.

**Reviewer Comment:**

*If this job is for the benefit of biologists, why not use transcripts as a reference? Because RBP binds to transcripts. And it's too simplistic for systems biologists, the author can refer to the POSTAR3 data.*

***Author Response: *** Our choice to focus on genomic coordinates was driven by the goal of providing a comprehensive resource for accessing RBP binding sites across the genome. While transcripts offer valuable context, our approach allows for flexibility in analyzing binding sites within different genomic regions and across multiple transcripts. However, we recognize the importance of incorporating transcript-level information and will explore integrating such data in future updates of the webserver to enhance the utility of our tool for systems biologists.
